# An initial experience with the use of whole body MRI for cancer screening and regular health checks

**DOI:** 10.1371/journal.pone.0206681

**Published:** 2018-11-21

**Authors:** So Yeon Lee, Hee Jin Park, Mi Sung Kim, Myung Ho Rho, Chul Hee Han

**Affiliations:** Department of Radiology, Sungkyunkwan University School of Medicine, Kangbuk Samsung Hospital, Seoul, Korea; McLean Hospital, UNITED STATES

## Abstract

**Objectives:**

We evaluated the utility of whole-body MRI (WB MRI) for cancer screening and other regular health evaluations.

**Methods:**

This retrospective study included 229 patients who underwent whole-body MRI as part of a routine health examination and cancer screening. The WB MRIs and radiologic reports were evaluated by a musculoskeletal radiologist, a neuroradiologist, and an abdominal radiologist. The consensus of their findings was characterized into three categories, as follows: suspicion of malignancy (category I); need for follow-up (category II); and no need for follow-up (category III). Any correlations between the abnormal findings and each study group were evaluated using the Mann-Whitney U test and chi-square test.

**Results:**

There were six category I lesions, among which two cases were found to involve malignancy. The most common category II findings were annular tears of the disc (14% of category II findings) and severe disc bulging or protrusion, followed by shoulder bursitis and uterine myoma. The most common category III finding was mild disc bulging or protrusion (47% of category III findings).

**Conclusions:**

WB MRI can be used in cancer screening and for regular health evaluations. WB MRI not only provides information about potential malignancy, but also provides information regarding nonmalignant abnormalities that require further evaluation.

## Introduction

Various diagnostic procedures, including ultrasound, computed tomography (CT), magnetic resonance imaging (MRI), positron emission tomography (PET) and X-ray, are used for cancer screening and regular health checks [1]. However, complete screening can become very time-consuming and inconvenient for patients if several different imaging modalities are required. Therefore, it would be most time efficient if a single imaging modality could provide information about multiple organs, or even the entire body. The intention of screening is to detect a wide range of diseases before they become clinically evident. In addition, screening should screen particularly for diseases in which early treatment improves disease-related morbidity and mortality [2]. Whole-body MRI (WB MRI) is proposed as a potential modality to evaluate the entire body with excellent spatial resolution and high sensitivity [3,4]. Whole-body MRI is particularly helpful in detecting metastases. Few studies have evaluated the use of WB MRI (not including a specialized MR technique such as MR mammography) for cancer screening and regular health checks in the general population [5]. Although Hegenscheid et al. [6] included WB MRI in their epidemiological study of the German population, they also performed cardiac MR and MR mammography. Further, Tarnoki et al. [7] reported that WB MRI was a useful method in cancer screening, even though their results were based on a small patient population (n = 22). Our study is the first to evaluate the utility of coronal and sagittal WB MRI for cancer screening and routine health exam in a large number of asymptomatic patients. The purpose of our study was not only to evaluate the usefulness of WB MRI for cancer screening, but also to assess whether it has other benefits in routine health maintenance.

## Materials and methods

### Case selection

The study was performed in compliance with HIPAA guidelines. It was approved by the institutional review board at Kangbuk Samsung hospital. The requirement for informed consent was waived by the institutional review board of Kangbuk Samsung Hospital for this retrospective study design. All patient data were fully de-identified. The study population consisted of 229 consecutive patients (including 139 (60%) men and 90 (40%) women) who underwent whole-body MRI as part of a routine health check at our institution between April 2013 and March 2014. Patients ranged in age from 37 to 73 years with a mean age of 52 (standard deviation of 7). All of the included patients are Korean. Patients were excluded if they had a known malignancy or anatomic changes due to a previous surgery.

### Magnetic resonance parameters

WB MRIs were acquired using a 1.5-T magnet MRI scanner (Signa HDxt, GE Healthcare, Milwaukee, WI, USA). Detailed sequences and imaging parameters are summarized in Table 1. Patients were placed in the supine position. The MRIs were performed in four parts anatomically, as follows: head and neck; trunk; thigh; and leg. All of these separate images were consolidated into a single image for each patient. We used the head & neck coil, 12-channeled body array coil and inherent coil of the gentry for imaging. The matrix sizes and fields of view varied by the body portions (Table 1). Three trained technicians performed all of the examinations in an identical manner. No contrast agents were used in this study.

**Table 1:** Imaging parameters for MR sequences.

Imaging parameter	Coronal T_1_ Fat suppression (3D SPGR)	Coronal T_2_ STIR	Whole-body sagittal T_2_ Fast spin echo
TR (msec)	7.6/6.3 (abdomen)	6000/2800 (abdomen)	4000
TE (msec)	3.6	35	110
TI (msec)	-	130	-
Flip angle (°)	15	180/90	90
Matrix size	300 X180	320 X 192	480 X 256
Field of view (cm)	160 X 44	160 X 44	90 X 10
Slice thickness (mm)	3	7	4
Inter-slice gap (mm)	0	1	0.5
Bandwidth (kHz)	83.33	62.50	41.67
Echo train length	9	9/12 (abdomen)	26
Signal average	1	1	4.0
Scan time (min:sec)	1:29/0:21/2:46/1:34	2:12/1:20/2:44/2:30	2:46/2:46

### Image analysis

A fellowship-trained musculoskeletal radiologist, neuroradiologist, and abdominal radiologist (with 14, 20, and 15 years of experience, respectively) independently read each image. Later, all three radiologists evaluated the WB MRIs and radiology reports in consensus. In the case of disagreements, the diagnosis was made by the consensus of at least two of the radiologists. The radiologists first evaluated whether the initial radiologic diagnoses were correct and whether any important findings had been omitted. They then classified the positive findings into three main categories. Category I included findings that strongly suggested malignancy. Category II included findings that suggested abnormalities requiring further evaluation, such as hydronephrosis, severe disc protrusion causing compression of the cauda equina (that manifested as redundant cauda equina above the lesion or myelopathy), and benign tumors containing complicated cystic lesions [8, 9]. A cervical neck lymph node was considered positive if it was >1 cm in short diameter. Category III included normal findings or findings commonly observed in asymptomatic patients, such as: normal variants, chronic brain lacunar infarcts, mild disc protrusion (below Lee grade 2), functional ovarian cysts, perineural cysts, simple bone cysts, and vertebral hemangioma [8].

### Statistical analysis

The incidence of incorrect or omitted reports was calculated. We classified the study population into two separate groups according to age and sex (< 52 years, ≥ 52 years given that 52 years was the mean age of the population). The prevalence of the positive findings according to age and sex was calculated using the Mann-Whitney U test. The chi-square test was used to analyze the relationship between positive findings of each category and patient characteristics. Statistical analyses were performed using the PASW software, version 18.0 (IBM, Armonk, NY, USA). *P*_*-*_values < 0.05 were considered statistically significant.

## Results

**Fig 1:**
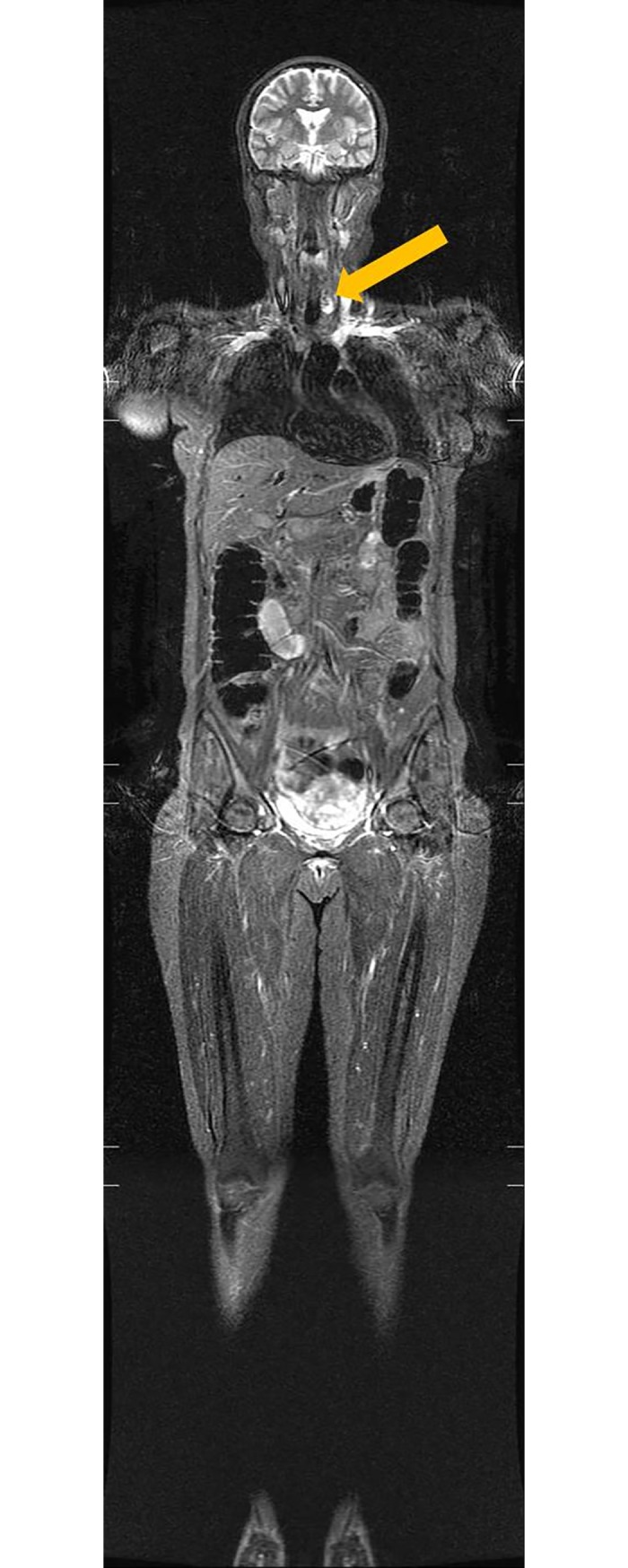
A 47-year-old woman who underwent a routine health examination. Coronal T*2*-weighted STIR image (TR/TE, 6000, 2800/35) revealed a 20-mm hyperintense nodule in the left portion of the thyroid gland (arrow). The initial radiologic diagnosis was benign thyroid nodule. Radiologist reviewers classified this lesion as category II.

**Fig 2:**
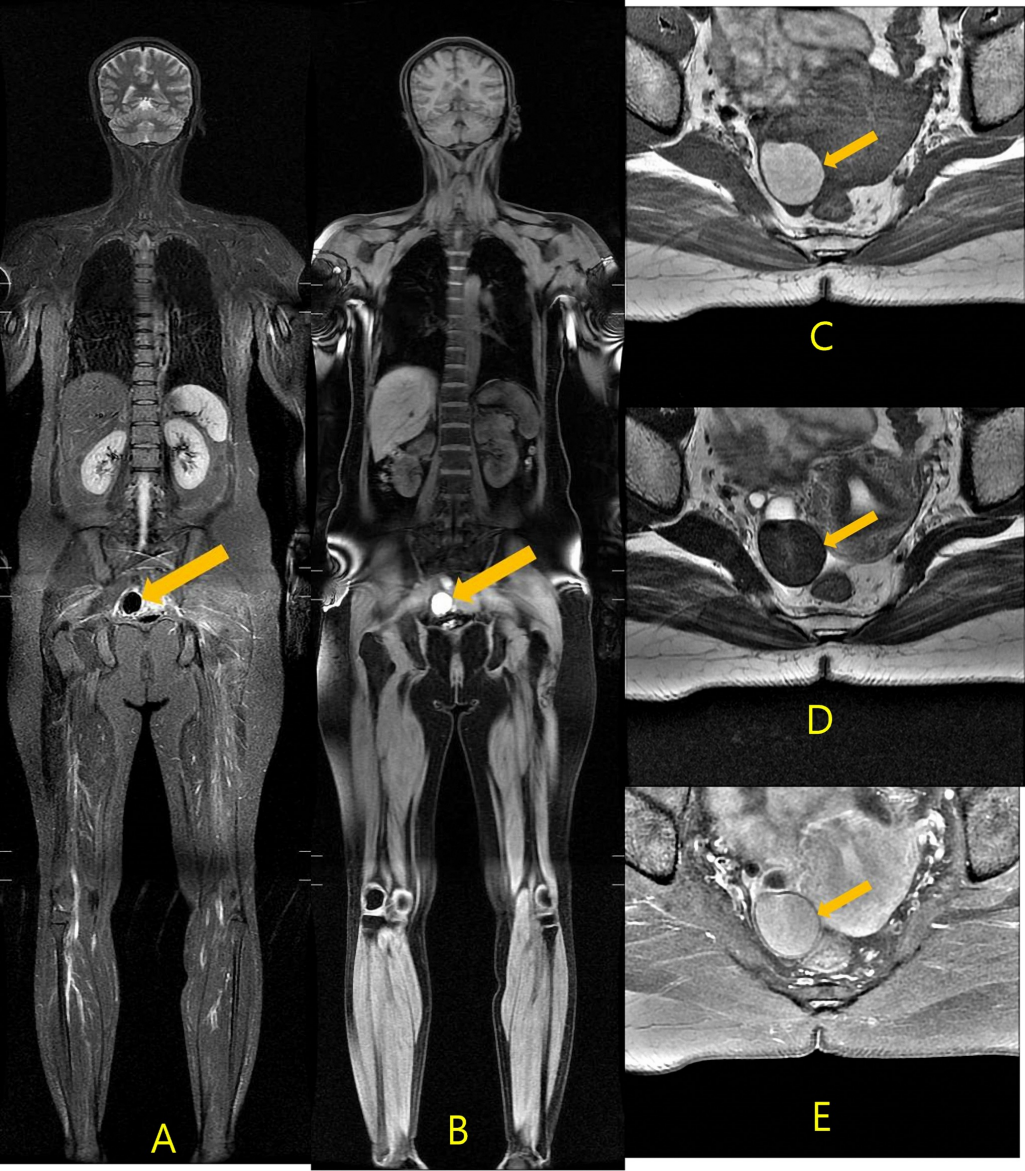
A 43-year-old woman who underwent a routine health examination. Coronal T_2_-weighted STIR image (TR/TE, 6000, 2800/35, A) and coronal T_1_ fat suppression SPGR (TR/TE, 7.6, 6.3/3.6, B) images revealed a well-defined, 3-cm hemorrhagic mass in the pelvic cavity (arrow). The initial radiologic diagnosis was endometrioma. Radiologist reviewers classified this lesion as category II. The follow-up axial MRI, including T_1_-weighted MR image (TR/TE, 600/9, C), T_2_-weighted MR image (TR/TE, 3700/100, D) and fat-saturated enhanced T_1_-weighted MR image (TR/TE, 800/10, E), revealed a 3-cm well-defined oval shaped mass at the right adnexa suggestive of an endometrial cyst (arrow).

**Fig 3:**
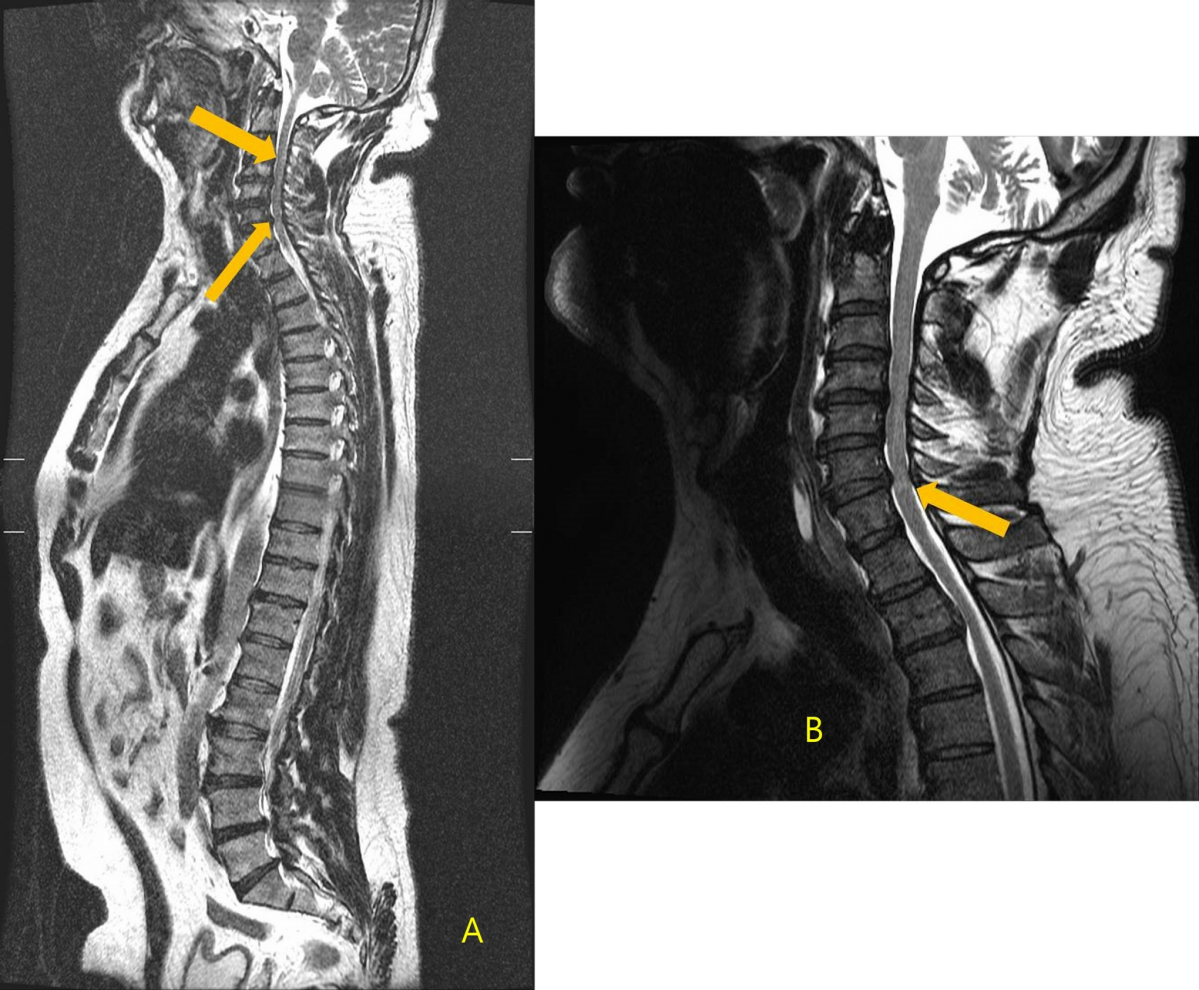
A 44-year-old man who underwent a routine health examination. A: Whole-body sagittal T_2_ fast spin echo (TR/TE, 4000/110) revealed a disc protrusion at C3-4 and C6-7 (arrows). A faint bright signal was seen in the spinal cord (thin arrow). The initial radiologic diagnosis was severe disc protrusion. Radiologist reviewers classified this lesion as category II.B: On the follow-up sagittal T_2_-weighted MR image (TR/TE, 300120) of the C-spine, definite myelopathy was seen in the spinal cord at the C6 level (arrow).

The number of lesions by patient age and sex are summarized in Table 2. Older patients (≥ 52 years) had significantly more lesions (per patient) than did younger patients (< 52 years; *p-*value = 0.03). However, there was no significant difference between the two age groups with regard to the incidence of positive findings (Table 3). There was also no significant difference in the number of lesions by sex (*p-*value > 0.05). In contrast, there was a significant difference between males and females in the incidence of category II and III lesions (*p*-value = 0.006). Men had more category III lesions than did women, while women had more category II lesions than did men. Overall, category III lesions were the most common, followed by category II lesions. The mean number of lesions per patient was 2.23. There were a total of 500 positive findings. The incidence of positive findings in each category is summarized in Table 4. We found six lesions belonging to category I. One of four detected renal masses was confirmed to be malignant on subsequent CT and MRI. The most common category II findings were annular tears of the disc (14% of total category II lesions), followed by severe disc bulging or protrusion, shoulder bursitis, and uterine myoma (Fig 1, Fig 2A–2E, Fig 3A and 3B). The most common category III finding was mild disc bulging or protrusion (47% of total category III lesions). One suspicious malignant lesion (tongue cancer) was first overlooked by the neuroradiologist. However, this lesion was ultimately detected by all three radiologists in consensus. Other lesions that were omitted included vertebral hemangiomas (two cases), simple bone cysts (four cases), a retention cyst in the maxillary sinus (one case), bone marrow edema (one case), a thyroid nodule (one case), and an arachnoid cyst (one case).

**Table 2:** Number of lesions per patient by age and sex (n = 229).

Patients	No lesion	1 lesion	2 lesions	3 lesions	4 lesions	5 lesions	6 lesions	Total	_*P*_-value
Age < 52	9 (8%)	33 (28%)	44 (37%)	20 (17%)	10 (8%)	4 (3%)	0 (0%)	120 (100%)	0.030
Age ≥ 52	7 (6%)	22 (20%)	35 (32%)	23 (21%)	15 (14%)	4 (4%)	3 (3%)	109 (100%)	
Male	12 (9%)	35 (25%)	47 (34%)	25 (18%)	15 (11%)	3 (2%)	2 (1%)	139 (100%)	0.135
Female	4 (4%)	20 (22%)	32 (36%)	18 (20%)	10 (11%)	5 (6%)	1 (1%)	90 (100%)	
Total	16 (7%)	55 (24%)	79 (34%)	43 (19%)	25 (11%)	8 (3%)	3 (1%)	229 (100%)	

**Table 3:** Positive findings (n = 500) by age and sex.

Patients	Category I	Category II	Category III	Total	_*P*_-value
Age < 52	3 (1)	97 (41)	137 (58)	237 (100)	0.990
Age ≥ 52	3 (1)	107 (41)	153 (58)	263 (100)	
Male	3 (1)	100 (35)	184 (64)	287 (100)	0.006
Female	3 (1)	104 (49)	106 (50)	213 (100)	
Total	6 (1)	204 (41)	290 (58)	500 (100)	

Note–Data in parenthesis are percentages

**Table 4:** Incidence of positive findings by category (n = 500).

Category	Findings	Number (%)	Frequency (%)
I	Renal mass	4 (1.7)	68
	Pancreas lesion	1 (0.4)	16
	Tongue mass	1 (0.4)	16
	Total	6	100
II	Annular tear	28 (12.2)	13.7
	Uterine myoma	23 (10.0)	11
	Severe disc bulging or protrusion	23 (10.0)	11
	Shoulder bursitis	23 (10.0)	11
	Bone marrow edema	14 (6.1)	6.9
	Hepatic nodule or mass	13 (5.7)	6
	Spondylolisthesis	11 (4.8)	5.4
	Synovitis (Hip, knee)	9 (3.9)	4.4
	GB stones	8 (3.5)	4
	Complex ovary cyst	6 (2.6)	3
	Hemorrhagic cyst	6 (2.6)	3
	Thyroid nodule	4 (1.7)	2
	Pancreatic duct dilatation	4 (1.7)	2
	Neural foraminal stenosis	4 (1.7)	2
	Compression fracture	4 (1.7)	2
	Meniscal injury	4 (1.7)	2
	Scoliosis	4 (1.7)	2
	GB polyps	3 (1.3)	1.5
	Cervical lymph nodes	2 (0.9)	1
	Diffuse thyroid abnormality	2 (0.9)	1
	Dilatation of biliary tree	2 (0.9)	1
	Hydronephrosis	1 (0.4)	0.5
	Lipoma	1 (0.4)	0.5
	Neurogenic tumor	1 (0.4)	0.5
	MCL injury	1 (0.4)	0.5
	OPLL	1 (0.4)	0.5
	SONK	1 (0.4)	0.5
	Aortic dissection	1 (0.4)	0.5
	Total	204	100
III	Mild disc bulging or protrusion	136 (59.4)	46.9
	Renal cyst	57 (24.9)	19.6
	Hepatic cyst	54 (23.6)	18.6
	Hemangioma-like lesion (vertebra)	11 (4.8)	3.8
	Simple bone cyst	9 (3.9)	3.1
	Retention cyst	8 (3.5)	2.8
	Perineural cyst	6 (2.6)	2
	Arachnoid cyst	3 (1.3)	1
	Pancreas cystic lesion	2 (0.9)	0.7
	Baker’s cyst	2 (0.9)	0.7
	Cerebromalacia	1 (0.4)	0.3
	Sacral meningocele	1 (0.4)	0.3
	Total	290	100

Note: Number = number of the lesion

(%) = percentage among each category

OPLL = ossification of the posterior longitudinal ligament

SONK = spontaneous osteonecrosis of the knee

## Discussion

Cancer screening requires systemic examination in order to detect unsuspected malignant disease [2]. In many countries, including South Korea, PET/CT is used for cancer screening because no other single imaging modality provides information about multiple organs or the entire body. However, PET/CT exposes patients to substantial radiation exposure because the effective dose is a combination of radiation from both PET and CT [10]. The effective dose of PET/CT is about 32 mSv. The lifetime attributable risk for cancer of this dose is up to 0.514% for a 20-year-old man [10]. Whole-body MRI can be considered in lieu of PET/CT for cancer screening. MRI has potential benefits over PET/CT, because the radiofrequency energy and magnetic fields that are used for MR imaging are not suspected to pose any biological risk [2,11,12]. WB MRI uses a small number of sequences, which minimizes the slice number and enables fast coverage of the entire body. The total scan time for WB MRI is comparable to that of specialized MR studies [13]. Many medical centers use WB MRI to evaluate metastases because it is more sensitive than PET/CT in the detection of bony metastases [13,14,15,16]. Hegenscheid et al. [6] reported that WB MRI is useful for epidemiological studies, such as in their study of 200 volunteers, because the image quality and examination time are acceptable. This group also reported that not only does WB MRI provide valuable information regarding disease prevalence in a general population, but also has reliable inter-observer agreement [6]. The main limitation of the study by Hegenscheid et al. is that their total scan time was prolonged because they also included cardiac MR and MR mammography. Therefore, the overall scan time was more than two hours [6]. One of the prerequisite conditions for cancer screening, however, is a reasonable scan time. The total scan time in our study did not exceed 20 minutes.

Most patients (65%) had two or fewer lesions, although older patients had many more lesions than younger patients did (Table 2). This age discrepancy is not unexpected because the incidence of disease generally increases with age. Category II lesions were found more commonly in women than they were in men. This difference may result from the fact that uterine myomas, complex ovarian cysts and hemorrhagic cysts, none of which are found in men, were included in class II. In contrast, class III lesions were found more commonly in men than they were in women. We cannot clearly explain this difference. We found six cases of lesions suspicious for malignancy (category I) among the 229 patients (2.6%). Two of these cases were pathologically confirmed to be malignant (0.8%). Mizuma et al. [17] reported that 4.76% of the general population required further radiologic or laboratory evaluation; 0.087% of 1,600 patients were confirmed to have malignant lesions in a study of sonographic screening for the detection of abdominal cancer. Our cancer detection rate was higher than that reported by Mizuma et al., although the two studies are not entirely identical. Almost every patient with a class I lesion underwent additional studies for a final diagnosis. However, we believe that WB MRI is valuable for screening because the primary goal of screening is the detection, rather than confirmation, of a fatal disease.

Among the 229 patients in our study, we identified approximately 200 category II lesions that required further evaluation. Detection of these cases highlights the main benefit of WB MRI screening over PET/CT screening. Category III lesions can be classified as incidental findings. An incidental finding is an asymptomatic lesion that is found while examining a patient for other reasons [18]. Mild disc protrusion was the most common lesion (47%), followed by renal and hepatic cysts (Table 4). Park et al. [18] reported the occurrences of spinal incidental findings during herniated disk disease evaluation of the L-spine with MRI. Their incidence of vertebral hemangioma was 1.5%, perineural cyst 2.1% and sacral meningocele 0.8%. We found similar incidences of 3.8%, 2% and 0.3%, respectively (Table 4).

WB MRI also has its disadvantages. First, it cannot provide information about detailed anatomic structures with three-dimensional sequences. Therefore, we were unable to evaluate subtle meniscal injuries and ligament injuries in the knees due to limited image resolution. The elbow was not included in the field of view because both arms were too far from the midline of the body. The lack of human resources, such as a musculoskeletal specialist (who can provide detailed information regarding WB MRI) is also a problem in many countries.

Although some lesions are known to be more easily detected with enhanced imaging, we did not use MRI contrast material because it is inappropriate for screening purposes. MRI contrast media can cause complications ranging from mild side effects to life-threatening events.

One of the limitations of our study was that WB MRI did not evaluate for all malignant diseases, such as breast cancers and small pituitary tumors. Furthermore, MRI has only fair accuracy in the assessment of air-tissue boundaries due to susceptibility artifacts; therefore, evaluation of lung and bowel lesions was also limited [2]. Another limitation is the inability to detect small (<6 mm) lung nodules on WB MRI. Furthermore, our study did not have a sufficient follow-up period. The interval between WB MRI and our retrospective study was less than 6 months for some patients. Finally, we did not evaluate the cost effectiveness of MRI or its effects on survival, as this was a preliminary study of cancer screening.

In conclusion, WB MRI can be considered as a suitable substitute for PET/CT in cancer screening. WB MRI also provides information about other nonmalignant abnormalities that require further evaluation and cannot be detected with PET/CT screening.

## Supporting information

S1 Data Set [Anonymized data (english)]. (XLSX)
